# Possible Role of 5-Hydroxytryptamine (5-HT) Receptor on Human Sperm Motility Regulation

**DOI:** 10.7759/cureus.49530

**Published:** 2023-11-27

**Authors:** Maya Omote, Yu Wakimoto, Hiroaki Shibahara

**Affiliations:** 1 Obstetrics and Gynecology, Hyogo Medical University School of Medicine, Nishinomiya, JPN

**Keywords:** sperm motility, serotonin, reproductive medicine, male factor infertility, in-vitro fertilization

## Abstract

The purpose of this study is to examine whether 5-hydroxytryptamine (5-HT, also known as serotonin) regulates human sperm motility, focusing on 5-HT receptors. Immunofluorescent staining revealed the existence of seven types of 5-HT receptors with a heterogeneous pattern of reactive sites. In detail, 5-HT_1B_, 5-HT_6_, and 5-HT_7_ were detected in the post-acrosomal and mid-piece regions. The 5-HT_2A_ and 5-HT_5A_ receptors were mainly localized in the equatorial segment. 5-HT_3A_ and 5-HT_4_ receptors were present in the neck and post-acrosomal regions. When examining the effects of 5-HT receptor antagonists on sperm motility, only the 5-HT_2A _receptor antagonist significantly reduced sperm motility. This suggests that the 5-HT_2A_ receptor may have a regulatory function in sperm motility. Eventually, progressive motility should be attenuated to penetrate the oocyte for fertilization. The current study indicated heterogenous expression patterns and plausible functions of 5-HT receptors in human sperm.

## Introduction

According to a World Health Organization (WHO) report [1], infertility is partly caused by the male factor. 5-HT is a known mood-stabilizing neurotransmitter in the brain [2]. 5-HT is synthesized from the essential amino acid tryptophan [3] and is distributed throughout the nervous system, gut, mast cells, platelets, neuroendocrine cells, and reproductive organs [4]. The 5-HT receptor proteins comprise seven families: 5-HT_1_, 5-HT_2_, 5-HT_3_, 5-HT_4_, 5-HT_5_, 5-HT_6_, and 5-HT_7_ [4,5]. Since 5-HT receptors are target molecules that regulate signaling such as Ca2+ signaling via Gq proteins and phospholipase C, which activates soluble intracellular adenylate cyclase, 5-HT has been proposed as a promising candidate for new drug design [6]. Many cases of male infertility are due to sperm dysfunction [1]. It is known that ejaculated sperm usually undergo an acrosome reaction in the female reproductive tract after achieving final fertilization competence (fertilization competence). In addition to sperm plasma, the female reproductive tract contains a variety of physiological and biological components. One of these components, 5-hydroxytryptamine (5-HT, also known as serotonin), was suggested to be a strong candidate for regulating sperm fertility.

In reproductive organs, 5-HT regulates gametogenesis, as well as hormone synthesis and secretion [6,7]. Several studies using human sperm have described the relationship between blood serotonin concentration and semen quality [8,9]. However, to our knowledge, there are few reports concerning 5-HT concentrations in seminal plasma and molecular effects on sperm function. Only animal studies have indicated that 5-HT induces contraction of the vas deferens and regulates testicular blood flow and testicular growth [10-12].

Furthermore, there are few reports of direct effects of 5-HT on sperm function [13]. Recently, however, it was reported that 5-HT stimulated hyperactivation and the acrosome reaction in hamster sperm [14]. Our group also observed that 5-HT improved success rates of in vitro fertilization, as well as hyperactivation rates of mouse sperm [15]. In human sperm experiments, Jiménez-Trejo et al. [16] demonstrated the presence of 5-HT_2A_ receptors, our knowledge only this research group has investigated the relationship of 5-HT to human sperm. Based on their findings, we intended to confirm the effect of 5-HT on sperm motility but did not obtain coincident results. Instead, 5-HT showed no effect on human sperm motility.

Then, we focused on the 5-HT receptor antagonists and examined their effects on human sperm motility. The results showed that only the 5-HT_2A_ receptor antagonist significantly reduced human sperm motility, suggesting that progressive motility should be attenuated to penetrate the oocyte for fertilization. It is thus concluded that the 5-HT_2A_ receptor plays an important role in sperm motility regulation at the fertilization site. 5-HT and related receptors may have different functions at several stages in the female reproductive tracts such as sperm transport, sperm-zona pellucida binding, and penetration of the oocytes.

## Materials and methods

Ethical approval, research subjects, and sample processing

This study was approved by the Ethics Review Committee of Hyogo Medical University (approval number: 2997). Procedures were in accordance with the ethical standards of the Committee Responsible for Human Experimentation and the 1964 Declaration of Helsinki. Thirty-nine sperm samples were collected from adult male patients or volunteers who visited the Hyogo Medical University Hospital Clinic between September 2019 and December 2021 and gave written informed consent. The age of the subjects ranged from 22 to 59 (34 ± 10.22) years (Table 1). One specimen was excluded because it had 0% sperm motility. The donated 38 semen was liquefied at room temperature (23-25°C) for 60 minutes and then incubated in Arctic Sperm Cryopreservation Medium (FUJIFILM Irvine Scientific Inc., Santa Ana, CA, USA) containing human serum albumin (HSA) at a minimum semen volume of 1.4 mL and a total sperm concentration of 1.6 million/mL, and sperm motility was adjusted to at least 30%.

**Table 1:** Condition of semen before adjustment Details of 39 samples; no. 9 was excluded due to 0% migration

No.	Age (years)	Volume (ml)	Motility (%)	Total number of sperm (×10^4^/ml)			Varicose veins in the testes anamnesis
1	29	3.3	63	4050			non
2	27	4.5	30	2700			non
3	44	4	31	2600			non
4	31	3.6	56	4950			non
5	39	3.2	64	11200			non
6	41	1.1	19	41000			non
7	44	2.8	13	14500			non
8	40	5.3	41	12950			non
9	45	1.5	0	9			non
10	50	2	67	4556			non
11	45	1	33	4400			non
12	43	4	57	7980			left
13	33	2	51	3774			non
14	33	0.8	90	1440			non
15	45	1.5	19	6750			non
16	38	3	33	594			non
17	35	3	71	7455			left
18	59	1	18	900			non
19	53	5	28	4900			non
20	46	4	26	2900			non
21	31	2.5	50	3500			non
22	47	2	42	14616			non
23	30	4	40	4320			non
24	37	4	61	4392			left
25	30	2	60	1800			non
26	32	4	33	400			non
27	22	4	38	4864			non
28	28	3	63	19494			non
29	29	1.7	63	7018			non
30	27	4.9	25	1941			non
31	29	1.7	21	780			non
32	28	2.7	52	13697			non
33	29	1.4	40	861			non
34	28	2.3	76	10952			non
35	27	3.1	94	46534			non
36	28	2.2	49	780			non
37	29	3	49	5817			non
38	28	1.5	65	11717			non
39	28	1.8	51	6730			non
Average	35	2.6	45	7687			

Estimation of 5-HT concentrations in seminal plasma

The 5-HT concentration in seminal plasma was measured using a 5-HT ELISA kit (ENZO Life Science, Farmingdale, NY, USA). Twelve samples were randomly selected from semen with sperm motility of 19-82.4%. The correlation was assessed by the r value (Excel program, Microsoft, Washington, USA).

Sperm preparation for motility assay

After thawing sperm materials, they were removed from the freezing tube using a transfer pipette, added to 6 mL of Multipurpose Handling Medium-Complete (MHM-C), supplemented with gentamicin and HSA (FUJIFILM Irvine Scientific Inc., Santa Ana, CA, USA), prepared in a 15-mL tube, and stirred gently without whisking. The sperm samples were centrifuged twice at 362 × g for five minutes to remove seminal plasma and cryopreservation medium. The sperm pellet was resuspended in 1000 μL of MHM-C and incubated for 60 minutes at 37°C to allow the sperm to swim up. Sperm motility was measured by the Sperm Motility Analyzing System (SMAS) at three minutes, 15 minutes, one hour, and two hours after the addition of 5-HT at concentrations of 100 µM or 10 µM. Each sample (4 µL) was subjected to analysis using a standard counting chamber that was 12 µm deep with a two-window specification (Leja, SC-12-01-C; Neuroscience, Inc., Tokyo, Japan). Semen samples that did not exhibit motility after thawing were excluded from the study.

Immunostaining of 5-HT receptors

After adding 6 mL of MHM-C to thawed semen samples, sperm were washed and centrifuged twice at 362 × g for five minutes to remove seminal plasma. The washed sperm were subjected to swim-up treatment in MHM-C for 60 minutes. Swim-up sperm were collected and centrifuged at 362 × g for five minutes and then resuspended in phosphate-buffered saline (PBS, 0.01 mol/L, pH 7.4; LSI Medience Corporation, Tokyo, Japan) and agitated by a vortex mixer for 10 seconds. Sperm were smeared on glass slides (Matsunami, Osaka, Japan), dried for 30 minutes, fixed in neutral buffered 10% formalin (FUJIFILM Wako Chemical Scientific, Osaka, Japan) for 15 minutes, and then washed twice with PBS for five minutes. The specimens were blocked with EzBlock Chemi solution (ATTO Corporation, Tokyo, Japan) for one hour to suppress non-specific reactions and then incubated with primary antibodies diluted with PBS in a humidified chamber for 24 hours at 4°C. The primary antibodies used were as follows: anti-5-HT_1B_ polyclonal rabbit IgG (C12009, 1:100; Assay Biotech, Fremont, CA, USA), anti-5-HT_2A_ polyclonal rabbit IgG (C12013, 1:100; Assay Biotech, Fremont, CA, USA), anti-5-HT_3A_ polyclonal rabbit IgG (bs-2126R, 1:100; Bioss Antibodies, Woburn, MA, USA), anti-5-HT_4_ polyclonal rabbit IgG (NBP1-78403, 1:100; Novus Biologicals, CO, USA), anti-5-HT_5A_ polyclonal rabbit IgG (bs-12055R, 1:20; Bioss Antibodies Bioss Antibodies, Woburn, MA, USA), anti-5-HT_6_ polyclonal rabbit IgG (23561-1-AP, 1:100; Proteintech, Rosemont, IL, USA), and anti-5-HT_7_ polyclonal rabbit IgG (G018, 1:300; Assay Biotech, CA, USA). Reference Pure IgG (1:100; Proteintech, Rosemont, IL, USA) was used as a negative control. After washing three times with PBS for five minutes, the specimens were incubated with a secondary antibody (1:200; Alexa Fluor 555-labeled donkey anti-sheep IgG, or 1:1000, Alexa Fluor 488-labeled goat anti-rabbit IgG) diluted in EzBlock Chemi solution for one hour at room temperature. The nuclei were stained with 4′,6-diamidino-2-phenylindole (Life Technologies, Carlsbad, CA, USA). The specimens were observed under a fluorescence microscope BX53 (Olympus Corporation, Tokyo, Japan). Experiments were repeated three times for 5-HT receptor antibodies.

Analysis of 5-HT receptor antagonists

For antagonist experiments, the following 5-HT receptor antagonists were used: 5-HT_1B_ (GR55562 dihydrochloride; Abcam, Cambridge, UK), 5-HT_2A_ (cyproheptadine hydrochloride; MedChemExpress, Monmouth Junction, NJ, USA), 5-HT_3_ (dolasetron mesylate hydrate; Sigma-Aldrich, Missouri, USA), 5-HT_4_ (GR113808; Abcam, Cambridge, UK), 5-HT_6 _(SB271046A; Sigma-Aldrich, Missouri, USA), and 5-HT_7_ (SB258719; Sigma-Aldrich, Missouri, USA). 5-HT receptor antagonists (100 μM) were added to sperm suspensions, and motility assays were performed using SMAS including parameters of straight-line velocity (VSL), curvilinear velocity (VCL), average path velocity (VAP), linearity (LIN), straightness (STR), amplitude of lateral head displacement (ALH), and beat-cross frequency (BCF) (Ver3.09-Ver3.12; DITECT Tokyo, Japan). The details were described in the previous section “Sperm preparation for motility assay.” The experiments were repeated four times using different donors.

Statistics

A one-way ANOVA was used for statistical analysis, and Tukey’s method was used for post-hoc analysis using EZR software (Saitama Medical Center, Jichi Medical University, Saitama, Japan). Statistical significance was set at p<0.05.

## Results

**Figure 1:**
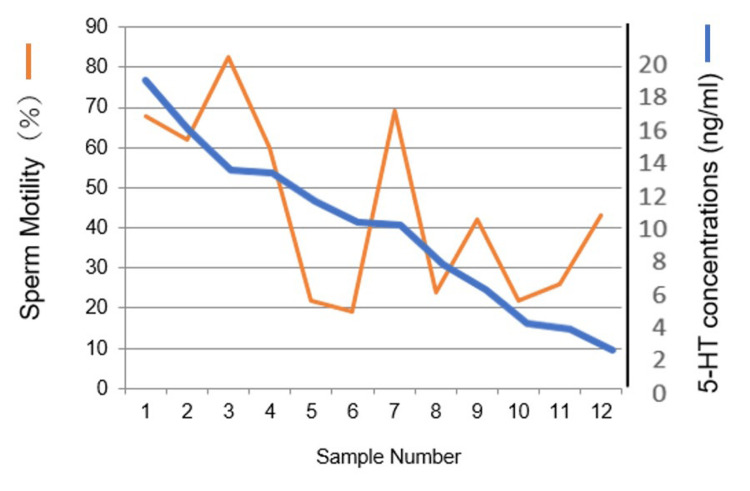
Relationship between 5-HT concentration in seminal plasma and sperm motility 5-HT concentration in seminal plasma correlated to sperm motility (r=0.605524)

**Figure 2:**
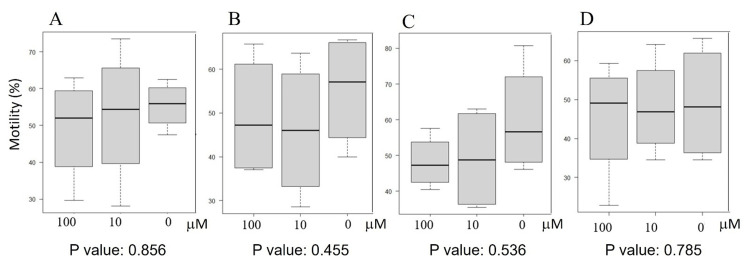
Effects of 5-HT on sperm motility at different incubation times Sperm motility was estimated after incubation with 5-HT for three minutes (A), 15 minutes (B), one hour (C), and two hours (D). Statistical analysis was carried out by the Kruskal-Wallis method. No significant difference was observed in any incubation times at concentrations of 10 mM or 100 mM. The concentrations used in this experiment were referred to in a previous paper [15].

First, we determined 5-HT concentrations in the seminal plasma because there is less data on this matter. The 5-HT concentration in the seminal plasma and sperm motility showed a significant positive correlation (Figure 1). When examined the effects of 5-HT on sperm motility, it did not affect sperm motility (Figure 2).

**Figure 3:**
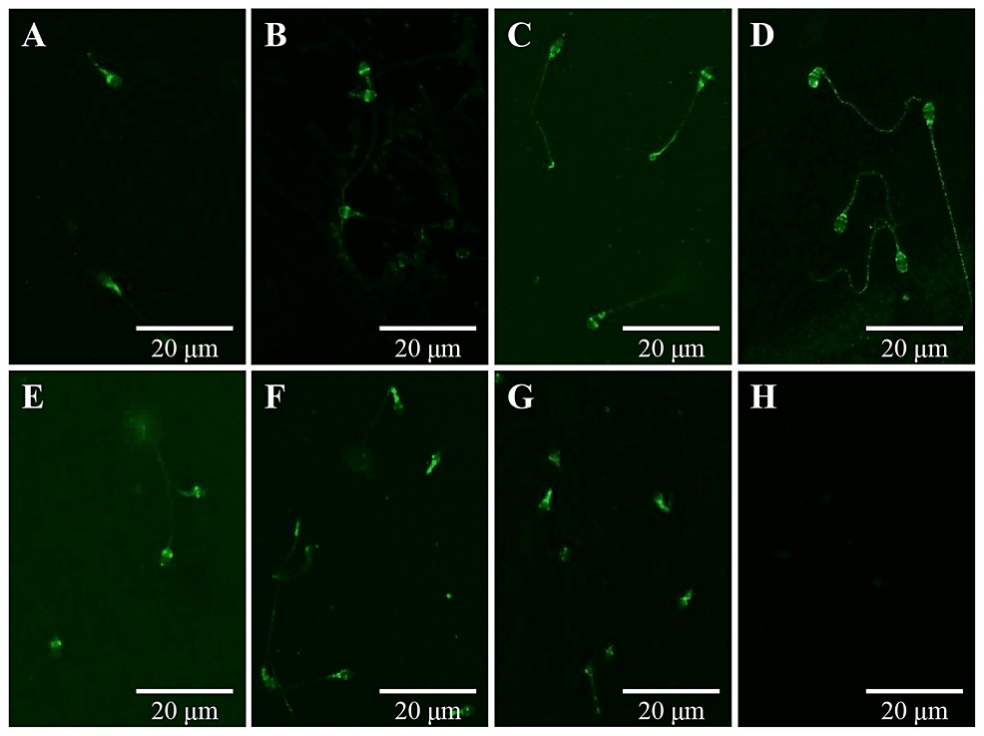
Detection of 5-HT receptors in human sperm by immunofluorescent staining A-H are represented for 5-HT_1B_, 5-HT_2A_, 5-HT_3A_, 5-HT_4_, 5-HT_5A_, 5-HT_6_, and 5-HT_7_ receptors and rabbit IgG (negative control), respectively. Experimental details are described in Materials and Methods.

As a next experiment, immunofluorescent studies were carried out to examine whether 5-HT receptors were localized on the sperm surface. The result showed that the 5-HT receptors were localized with heterogeneous patterns (Figure 3).

Of the seven types of 5-HT receptors, 5-HT_1B_, 5-HT_6_, and 5-HT_7_ reacted to the post-acrosomal and mid-piece regions (Figure 3A, 3F, 3G), 5-HT_2A_ and 5-HT_5A_ were detected in the equatorial segment (Figure 3B, 3E), and 5-HT_3A_ and 5-HT_4 _were mainly localized in the neck and post acrosomal regions with weak signals in the tail region of sperm (Figure 3C, 3D).

**Figure 4:**
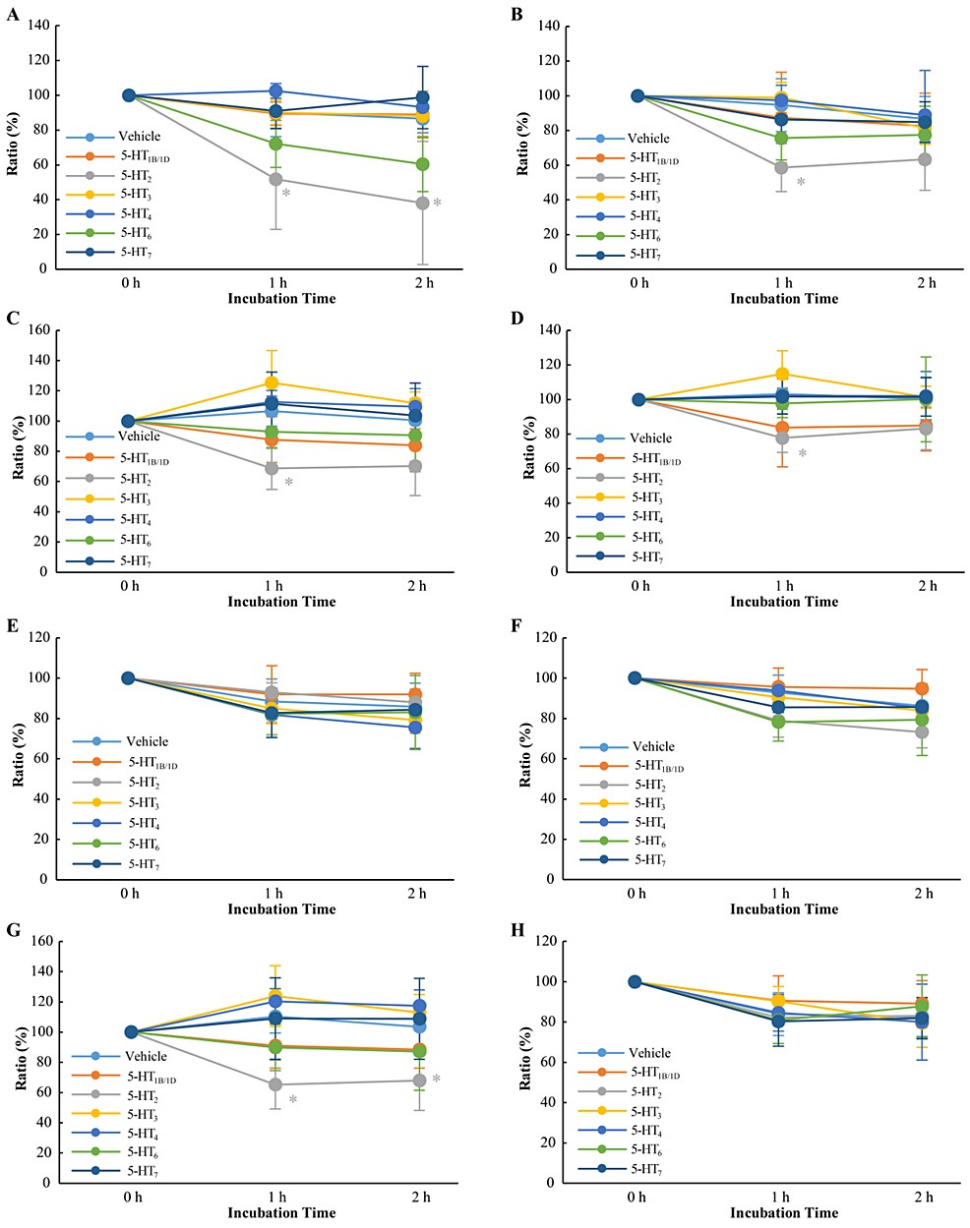
Effects of 5-HT receptor antagonist on sperm motility kinetics A: sperm motility, B: straight-line velocity (VSL), C: curvilinear velocity (VCL), D: average path velocity (VAP), E: linearity (LIN), F: straightness (STR), G: amplitude of lateral head displacement (ALH), and H: beat-cross frequency (BCF). The asterisk in “G” indicates a significant decrease (P<0.05).

Figure 4 shows the effects of 5-HT receptor antagonists on sperm motility kinetics. Only the 5-HT_2A_ receptor antagonist significantly reduced sperm motility after incubation for one and two hours (P<0.05). This receptor antagonist also reduced VSL, VCL, and VAP (Figure 4B-4D). The ALH also significantly decreased (Figure 4G). However, the 5-HT_2A_ receptor antagonist did not affect LIN, STR, or BCF (Figure 4E, 4F, 4H). Other 5-HT receptor antagonists did not affect sperm motility kinetics.

## Discussion

In WHO assessments [1], male factors are significant etiologic factors in infertile couples. Analysis of sperm fertilization mechanisms is contributing to the treatment of male infertility. The current study is focusing on 5-HT receptors present in human sperm. In order to ascertain their contribution to fertilization, the relationship between sperm motility and 5-HT receptors was examined.

Initially, we estimated 5-HT concentrations and determined the relationship between sperm motility and 5-HT concentration in individual seminal plasma and obtained high co-efficiency (r=0.605524). Although positive effects of 5-HT on sperm motility were expected, the addition of 5-HT did not show any influence, as shown in Figure 2. This suggests that 5-HT receptors are saturated by 5-HT and reach the accelerated stage of sperm motility in the ejaculated and liquefied sperm. This status is plausibly reasonable because progressive movement is necessary to reach the fertilization site in female reproductive tracts.

We detected seven types of 5-HT receptors from an immunofluorescence study. The results showed that the 5-HT receptor was located in a heterogeneous pattern of reactive sites, as shown in Figure 3. Future studies are needed to determine whether the localization of the 5-HT receptor is related to individual sperm function. Western blotting analysis represented multiple bands even in each receptor, suggesting that sperm 5-HT receptors have high molecular heterogeneity as well as distribution. 5-HT receptors generally possess many heterologous variants including splicing variants and edited variants as well as genetic variants [17,18]. The heterogenous pattern of the sperm 5-HT receptors is plausible in that there are various modifications of 5-HT receptors. Precise analysis will be needed using different epitope-recognizing antibodies for each 5-HT receptor.

The current study was designed to focus on 5-HT receptor inhibition because the addition of 5-HT had not affected sperm motility. Among the seven types of antagonists tested, only the 5-HT_2A_ receptor antagonist reduced sperm motility, including some parameters such as VSL, VCL, VAP, and ALH (Figure 4). The result strongly suggests that the 5-HT_2A _receptor plays an important role in sperm motility regulation. The signal transduction pathway of sperm motility was plausibly blocked via the antagonist so that sperm motility was regulated downward.

It is known that after migration to the fallopian tube, sperm primarily bind to the oocyte zona pellucida and induce an acrosome reaction [19]. At the first step of fertilization, a sperm protein located in the equatorial segment binds to the oocyte cell membrane [20]. During this process, it is considered that sperm bind to the oocyte and reduce motility to facilitate fertilization. Further movement is not needed and instead may interfere with fertilization. During sperm transport toward the upper portion of the female reproductive tracts, 5-HT in the seminal plasma may be reduced. Accordingly, motility promotion signals by 5-HT should also be attenuated. This condition is preferred for sperm to penetrate the oocytes. In physiological environments, cellular signals via HT_2A_ are reduced by lower concentrations of 5-HT. As another possibility, as-yet-undefined 5HT_2A_ inhibitors may suppress sperm motility.

Sperm fertilizing capacity including motility reduction may be controlled by 5-HT in the microenvironments of female reproductive tracts. Although the mechanism by which the blocking of the 5-HT_2A_ receptor is linked to sperm motility is still unknown, supplementation with a 5-HT_2A_ antagonist in an IVF medium may be able to promote fertilization by keeping the sperm on the oocyte surface.

There are some limitations to the present study. The first is that the abstinence period for sample collection ranged from two to five days, but the time of collection was not constant, such as in the morning or afternoon. The second is the setting of inhibitor concentrations in the experimental reagents. Although low concentrations (1, 10, and 100 μM) were used in the preliminary experiment, it was at the 100 μM concentration that a decrease in sperm motility rate was observed. Therefore, 100 μM was set, but this concentration was also set based on the pharmacokinetic concentration, which is the physiological concentration in the human body. Experiments were conducted with reference to previous studies [15].

## Conclusions

This study revealed the presence of seven types of 5-HT receptors in human sperm. Among them, 5-HT_2A_ receptors showed functional involvement in motility speed. Future studies should clarify the mechanism of 5-HT action involved in sperm function and whether 5-HT-related components improve male fertility.

5-HT is synthesized from tryptophan, an essential amino acid, and is a well-known neurotransmitter in the brain. 5-HT acts via receptors in human sperm. In the future, 5-HT may be useful in the treatment of male infertility.

The 5-HT_2A_ receptor is also the target of drugs for psychiatric symptoms of schizophrenia and Parkinson's disease, and this paper may provide a possible avenue for the treatment of male infertility by using an existing drug with an established safety profile for human effects.
